# Three-dimensional fractal dimension and lacunarity features may noninvasively predict TERT promoter mutation status in grade 2 meningiomas

**DOI:** 10.1371/journal.pone.0276342

**Published:** 2022-10-20

**Authors:** So Yeon Won, Jun Ho Lee, Narae Lee, Yae Won Park, Sung Soo Ahn, Jinna Kim, Jong Hee Chang, Se Hoon Kim, Seung-Koo Lee

**Affiliations:** 1 Department of Radiology, Kangbuk Samsung Hospital, Sungkyunkwan University School of Medicine, Seoul, Korea; 2 Department of Radiology and Research Institute of Radiological Science, Yonsei University College of Medicine, Seoul, Korea; 3 Department of Computer and telecommunication Engineerings, Yonsei University, Wonju, Korea; 4 Department of Nuclear Medicine, Wonju Severance Christian Hospital, Yonsei University Wonju College of Medicine, Wonju, Korea; 5 Department of Neurosurgery, Yonsei University College of Medicine, Seoul, Korea; 6 Department of Pathology, Yonsei University College of Medicine, Seoul, Korea; Goethe University Hospital Frankfurt, GERMANY

## Abstract

**Purpose:**

The 2021 World Health Organization classification includes telomerase reverse transcriptase promoter (TERT*p*) mutation status as a factor for differentiating meningioma grades. Therefore, preoperative prediction of TERTp mutation may assist in clinical decision making. However, no previous study has applied fractal analysis for TERTp mutation status prediction in meningiomas. The purpose of this study was to assess the utility of three-dimensional (3D) fractal analysis for predicting the TERTp mutation status in grade 2 meningiomas.

**Methods:**

Forty-eight patients with surgically confirmed grade 2 meningiomas (41 TERTp-wildtype and 7 TERTp-mutant) were included. 3D fractal dimension (FD) and lacunarity values were extracted from the fractal analysis. A predictive model combining clinical, conventional, and fractal parameters was built using logistic regression analysis. Receiver operating characteristic curve analysis was used to assess the ability of the model to predict TERTp mutation status.

**Results:**

Patients with TERTp-mutant grade 2 meningiomas were older (P = 0.029) and had higher 3D FD (P = 0.026) and lacunarity (P = 0.004) values than patients with TERTp-wildtype grade 2 meningiomas. On multivariable logistic analysis, higher 3D FD values (odds ratio = 32.50, P = 0.039) and higher 3D lacunarity values (odds ratio = 20.54, P = 0.014) were significant predictors of TERTp mutation status. The area under the curve, accuracy, sensitivity, and specificity of the multivariable model were 0.84 (95% confidence interval 0.71–0.93), 83.3%, 71.4%, and 85.4%, respectively.

**Conclusion:**

3D FD and lacunarity may be useful imaging biomarkers for predicting TERTp mutation status in grade 2 meningiomas.

## Introduction

The major change in the updated 2021 World Health Organization (WHO) classification of meningiomas is the integrated role of molecular markers to diagnose grade 3 (anaplastic) meningiomas [1]. In the 2021 WHO classification, mutation of the telomerase reverse transcriptase promoter (TERTp) and homozygous deletion of cyclin-dependent kinase inhibitor (CDKN) 2A/B upgrade grade 1 and grade 2 meningiomas to grade 3, even in the absence of markedly elevated mitotic activity (≥20 mitoses per 10 high-power fields [HPFs]) or frank anaplasia (resembling carcinoma, high-grade sarcoma, or melanoma) [2]. This is because the histologic grading criteria alone are unable to stratify prognosis in meningioma, and TERTp mutation and CDKN2A/B homozygous deletion are independent prognostic markers in grade 1 and grade 2 meningiomas [3–5].

Telomerase reverse transcriptase (TERT) is a catalytic enzyme that regulates stability and the length of telomere ends of chromosomes; therefore, it plays a vital role in the cellular aging process [6]. Activating mutations in its promoter, such as C228T and C250T, promote cellular immortalization, cancer development, and cancer progression of affected cells [7]. The reported incidences of TERTp mutation are approximately 1%, 6%, and 14%, respectively, in WHO grade 1, 2, and 3 meningiomas, according to the 2016 WHO classification [3]. Meningiomas with TERTp mutation have a higher rate of malignant transformation and are associated with a shorter time to recurrence and a lower overall survival rate than those without TERTp mutation [4,8,9]. Therefore, in addition to predicting prognosis, preoperative prediction of TERTp mutation in meningiomas may assist in clinical decision making by allowing clinicians to plan a more aggressive surgical approach.

Fractal features are model-based quantitative image features that characterize the geometric complexity of an object using mathematical approaches. Fractal dimension (FD) and lacunarity are the two parameters in fractal analysis. FD is a non-integer value that represents the intrinsic structure of an object; an increase in FD reflects an increase in geometric chaos. Lacunarity measures translational or rotational invariance and the degree of gappiness [10]. These features enable quantification of the structural complexity of an object, which is difficult to assess by traditional Euclidean geometry. Recent studies on fractal analysis have shown promising results in the field of neuro-oncology, including in differentiating meningioma grade [11,12], differentiating glioblastoma from central nervous system lymphoma [13], and predicting survival in glioblastoma [14,15]. However, to the best of our knowledge, no previous study has applied fractal analysis for TERTp mutation status prediction in meningioma.

We hypothesized that the TERTp mutation status in meningiomas may be reflected by geometrical complexity, which can be quantified by fractal features. The purpose of this study was to assess the utility of three-dimensional (3D) fractal analysis for predicting TERTp mutation in grade 2 meningiomas.

## Materials and methods

### Patient population

The requirement for informed consent for this retrospective study was waived by the Severance Institutional Review Board. Fifty patients with grade 2 meningiomas with known TERTp mutation status, who underwent preoperative magnetic resonance imaging (MRI) in our institution between August 2017 and July 2021 were retrospectively reviewed. Patients with a history of gamma knife surgery or tumor embolization before MRI were excluded (n = 2). Therefore, a total of 48 patients were included in the analysis.

### MRI protocols

Preoperative MRI was performed using a 3-T MRI scanner (Achieva, Philips Medical Systems, Best, The Netherlands) with an eight-channel sensitivity-encoding head coil. The preoperative MRI protocol included T1-weighted (repetition time [TR]/echo time [TE] 2000/10 ms; field of view [FOV], 230 mm; section thickness, 5 mm; matrix, 320 × 198), T2-weighted (TR/TE 3000/80 ms; FOV, 240 mm; section thickness, 5 mm; matrix, 256 × 256), T2-weighted fluid-attenuated inversion recovery (TR/TE 10,000/125 ms; inversion time, 2500 ms; FOV, 240 mm; section thickness, 5 mm; matrix, 256 × 256), and postcontrast T1-weighted (T1C) (TR/TE, 2000/10 ms; FOV, 250 mm; section thickness, 2 mm; matrix, 256 × 256) images. T1C images were acquired after administration of 0.1 mL/kg of gadolinium-based contrast material (Gadovist; Bayer).

### Pathologic diagnosis and molecular classification

Pathological diagnosis was performed by two neuropathologists in consensus using the 2016 WHO criteria [2]. The diagnostic criteria for WHO grade 2 meningioma, include 4–19 mitoses per 10 HPFs, the presence of brain invasion, and at least three of the following features: hypercellularity, “sheet-like” growth, large and prominent nucleoli, spontaneous necrosis, and small cells. The mitotic count was determined using the mitotic marker phosphohistone H3, and the mitotic index was measured by counting the number of unequivocal mitotic figures per 10 consecutive HPFs (×400) containing the most mitotically active areas. The Ki-67 labeling index, which represents the percentage of Ki-67- antigen positive cells, was estimated. TERTp mutation was determined using a pyrosequencing assay, and C228T and C250T mutations were analyzed as described previously [3,16].

### Qualitative imaging analysis

Conventional imaging findings [12,17–19], including tumor location (skull base or non-skull base), heterogenous enhancement, capsular enhancement, and presence of necrosis, were independently evaluated by two neuroradiologists (S.Y.W with 6 and Y.W.P with 9 years of experience) who were blinded to the clinical and histopathologic information. In cases of disagreement, a consensus was reached after discussion. Details of the definition of conventional imaging findings are shown in S1 File.

### Tumor segmentation

Tumor segmentation was performed using 3D slicer (version 4.11.0; http://slice.org) by a neuroradiologist (Y.W.P with 9 years of experience) who was blinded to the clinical and histopathological information. Regions of interests were semi-automatically drawn on every tumor slice on T1C images with threshold- and edge-based algorithms. Except for gross cystic, necrotic, and hemorrhagic portions, segmentation was performed by referring to conventional T1-weighted and T1C images. For assessment of interobserver agreement, randomly selected images from 30 patients were independently segmented by another neuroradiologist (S.S.A with 13 years of experience).

### Fractal analysis

The 3D FD and lacunarity values were calculated from the segmented masks using box-counting algorithms using Python [20]. The number of boxes including a part of the 3D binary mask was changed with respect to different box sizes to calculate the 3D FD. The squares of the coefficients of variation values of multiple boxes including a part of the 3D binary mask were averaged to compute the 3D lacunarity [21]. Because the optimal box size was unknown, different 3D box sizes (ranging from 21 to 27 isotropic voxels) were used to compute both FD and lacunarity. The mean FD and lacunarity were calculated for each patient. Details of FD and lacunarity calculations are available in Supplemental Material S2. Details of tumor surface regularity are also available in Supplemental Material S2.

### Statistical analysis

The clinical and imaging characteristics according to TERTp mutation status were compared using the chi-square test for categorical variables and the independent samples t-test or Mann–Whitney U test for continuous variables, according to normality. Interobserver agreement for conventional imaging findings was assessed using Cohen’s kappa index [22]. Interobserver agreement of fractal parameters was evaluated using two-way interclass correlation.

Univariable and multivariable logistic analyses were performed. The multivariable analysis was performed using significant variables in the univariable analysis (P < 0.05) with backward elimination according to the likelihood ratio and a variable selection criterion of P < 0.05. To detect multicollinearity between variables, a variance inflation factor was used. All variables included in the multivariable analysis showed variation inflation factors less than 10. Receiver operating characteristic (ROC) curve analysis to assess the accuracy of the model in predicting TERTp mutation status was performed by calculating the area under the curve (AUC). All statistical analyses were performed using the statistical software R (version 3.5.1). Univariable and multivariable logistic analyses were performed by using glm function of the R base package. P < 0.05 was considered statistically significant.

## Results

**Fig 1:**
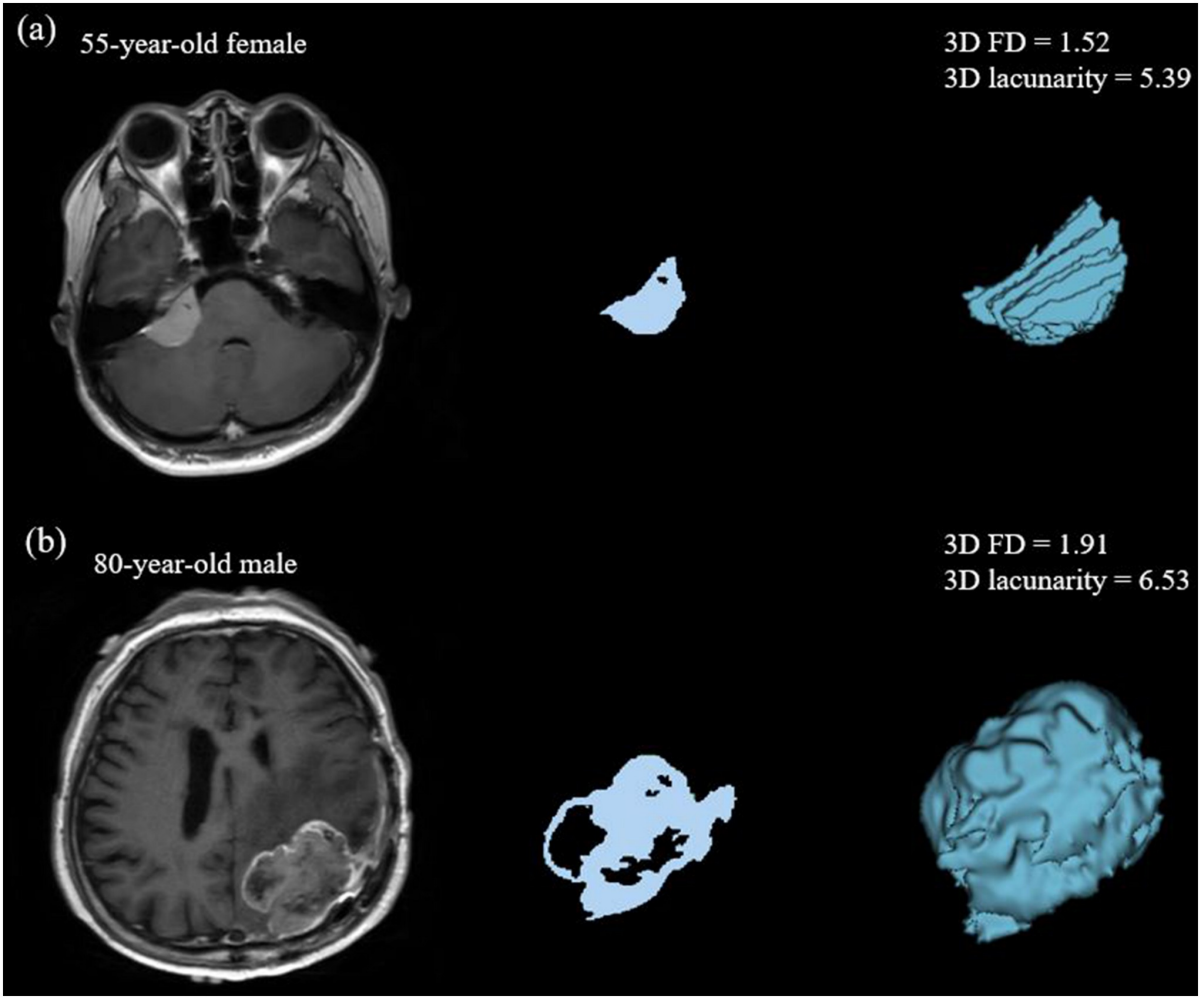
Representative cases of fractal parameters in grade 2 meningiomas according to TERTp mutation status. (a) Images of a 55-year-old female with TERTp wildtype grade 2 meningioma reveals an enhancing tumor in the right cerebellopontine angle on postcontrast T1-weighted image with a major axis of 2.9 cm. The 3D FD and 3D lacunarity were 1.52 and 5.39, respectively. (b) Images of an 80-year-old male with TERTp mutant grade 2 meningioma reveals an enhancing tumor in the left parietal convexity on T1C with a major axis of 5.3 cm. The 3D FD and 3D lacunarity were 1.91 and 6.53, respectively. FD = Fractal dimension, TERTp = telomerase reverse transcriptase promoter, T1C = Postcontrast T1 weighted image.

The clinical, pathological, and imaging characteristics of the 48 patients (25 women and 23 men; mean age, 58.2 ± 15.0 years) are summarized in Table 1. There were 41 (82.9%) and 7 (17.1%) patients with TERTp-wildtype and TERTp-mutant grade 2 meningiomas, respectively. Representative cases of TERTp-wildtype and mutant tumors are shown in Fig 1.

**Table 1:** Clinical, pathological, and imaging characteristics according to TERTp mutation status in grade 2 meningiomas.

Variables	TERTp wildtype (n = 41)	TERTp mutant (n = 7)	P-value*
**Clinical findings**			
Age (years)	56.29 ± 14.95	69.57 ± 10.33	0.029
Sex			0.549
Male	20 (48.8)	3 (42.9)	
Female	21 (51.2)	4 (57.1)	
Type of tumorumor			0.480
Primary tumor	38 (92.7)	6 (85.7)	
Recurrent tumor	3 (7.3)	1 (14.3)	
**Pathologic findings**			
Mitosis number	5.17 ± 1.88	6.93 ± 4.55	0.080
Ki-67 labeling index	5.05 ± 2.20	10.21 ± 7.06	< 0.001
**Conventional imaging findings**			
Skull base location	3 (7.3)	1 (14.3)	0.480
Heterogeneous contrast enhancement	19 (46.3)	6 (85.7)	0.062
Capsular enhancement	5 (12.2)	0 (0)	0.438
Presence of necrosis	14 (34.1)	2 (28.6)	0.571
Cystic change	8 (19.5)	1 (14.3)	0.608
Skull hyperostosis	10 (24.4)	3 (42.9)	0.278
Skull invasion	7 (17.1)	3 (42.9)	0.147
Max diameter (cm)	4.82 ± 1.48	6.15 ± 1.33	0.031
**Fractal parameters**			
3D FD	1.74 ± 0.16	1.88 ± 0.16	0.026
3D lacunarity	5.51 ± 0.41	6.01 ± 0.64	0.004

FD = Fractal dimension, TERTp = telomerase reverse transcriptase promoter.

Data are presented as mean ± SD or numbers of patients (%).

* Calculated from Student t test or Mann Whitney’s test for continuous variables and chi-square test or Fisher’s exact test for categorical variables.

### Interobserver agreement for qualitative and quantitative imaging analyses

The interobserver agreement for qualitative and quantitative imaging parameters was excellent (κ range, 0.872–0.972 and intraclass correlation coefficient range, 0.872–0.908) (S1 Table).

### Clinical and imaging parameters for predicting TERTp mutation status

**Fig 2:**
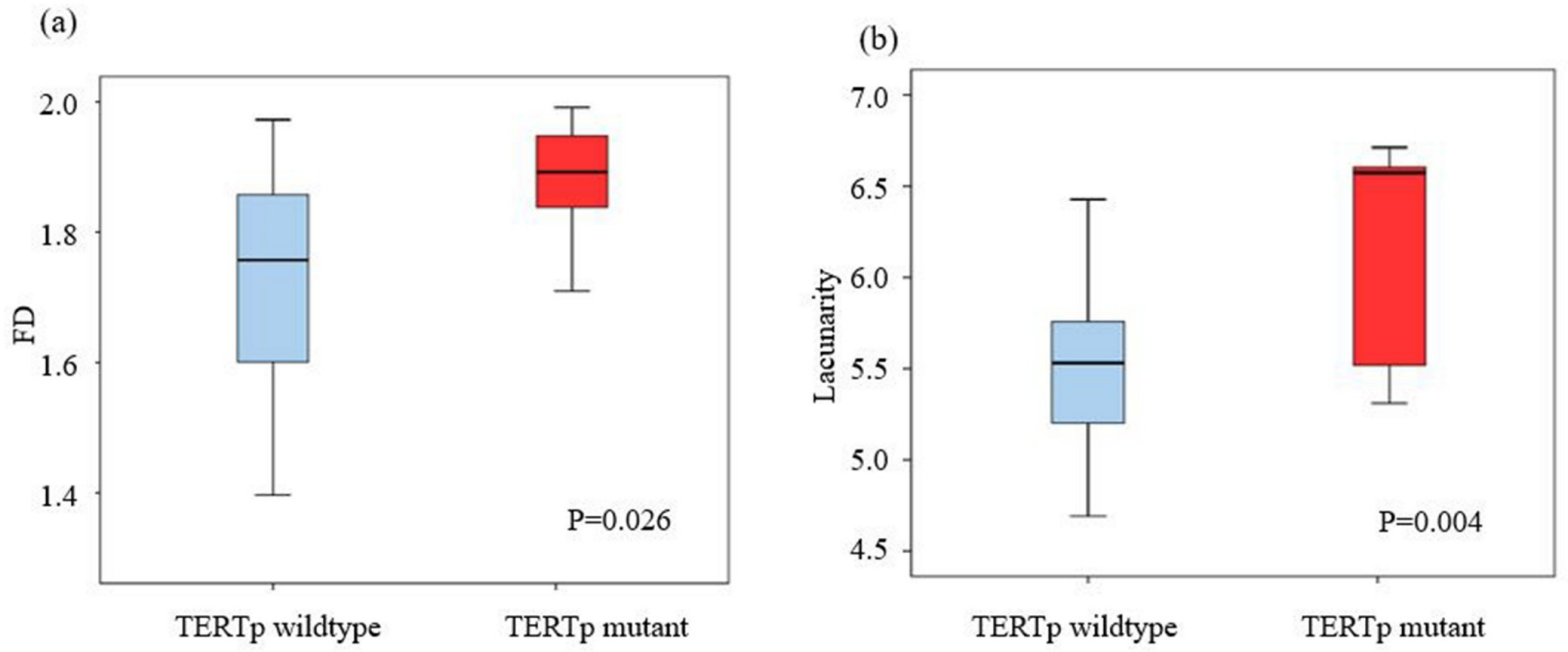
Boxplot representations. (a) 3D FD and (b) 3D lacunarity according to the TERTp mutation status. FD = Fractal dimension, TERTp = telomerase reverse transcriptase promoter.

With regard to clinical and pathological parameters, patients with TERTp-mutant meningiomas were older (69.6 years vs 56.3 years, P = 0.029) and had a higher Ki-67 labeling index (10.2 vs 5.1, P < 0.001) than those with TERTp-wildtype meningiomas. There were no statistically significant differences in other clinical parameters between the two groups. With regard to imaging parameters, TERTp-mutant meningiomas had a larger maximum diameter than TERTp-wildtype meningiomas (6.1 cm vs 4.8 cm, P = 0.031). With regard to fractal parameters, TERTp-mutant meningiomas had higher FD (1.9 vs. 1.7, P = 0.026) and lacunarity (6.0 vs. 5.5, P = 0.004) values than TERTp-wildtype meningiomas. Boxplots of 3D FD and lacunarity values according to TERTp mutation status are shown in Fig 2.

**Fig 3:**
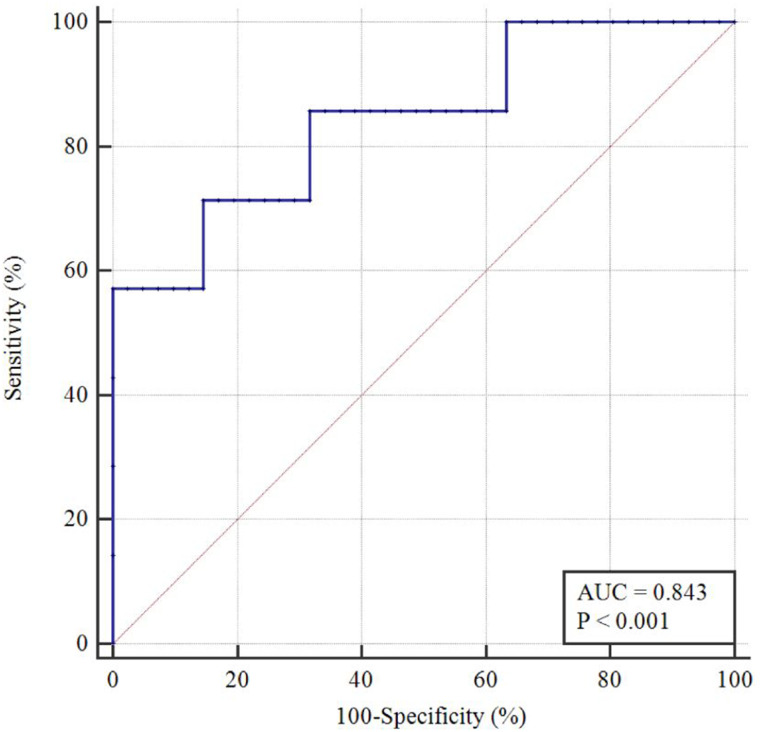
Receiver operating characteristic (ROC) curves of the multivariate logistic models to predict the TERTp mutation status. TERTp = telomerase reverse transcriptase promoter.

In the univariable logistic analysis, older age (odds ratio [OR] = 1.08, P = 0.042), larger maximum diameter (OR = 1.07, P = 0.042), higher 3D FD values (OR = 43.32, P = 0.046), and higher 3D lacunarity values (OR = 12.55, P = 0.011) were significant predictors of TERTp mutation (Table 2). In the multivariable logistic analysis, 3D FD (OR = 32.5, P = 0.039) and 3D lacunarity (OR 20.5, P = 0.014) values were independent predictors of TERTp mutation status. Fig 3 shows the ROC curves of the multivariable model for predicting TERTp mutation. The AUC, accuracy, sensitivity, and specificity of the multivariable model were 0.84 (95% confidence interval 0.71–0.93), 83.3%, 71.4%, and 85.4%, respectively.

**Table 2:** Univariable logistic analysis for clinical, pathological, and imaging predictors of TERTp mutation status.

	Univariable		Multivariable	
	OR (95% CI)	P-value	OR (95% CI)	P-value
**Clinical findings**				
Age (years)	1.08 (1.00–1.16)	0.042	-	-
Female sex	1.27 (0.25–6.40)	0.772		
Recurrent tumor	2.11 (0.19–23.78)	0.545		
**Imaging findings**				
Skull base location	2.11 (0.19–23.78)	0.545		
Heterogeneous contrast enhancement	6.95 (0.77–62.96)	0.085		
Capsular enhancement *	-	NA		
Presence of necrosis	0.77 (0.13–4.49)	0.773		
Cystic change	0.69 (0.07–6.55)	0.745		
Skull hyperostosis	2.33 (0.44–12.20)	0.319		
Skull invasion	3.64 (0.66–20.02)	0.137		
Max diameter (cm)	1.07 (1.00–1.15)	0.042	-	-
**Fractal parameters**				
3D FD	43.32 (1.16–162.18)	0.046	32.50 (2.83–89.39)	0.039
3D lacunarity	12.55 (1.79–87.90)	0.011	20.54 (1.86–227.12)	0.014

FD = Fractal dimension, TERTp = telomerase reverse transcriptase promoter, OR = Odds ratio.

ln (p/1-p) = -1.768 + 3.482 FD + 3.022 Lacunarity.

* Logistic analysis could not be performed due to complete separation.

## Discussion

This study examined the ability of 3D fractal analysis to predict TERTp mutation in grade 2 meningiomas. Our results enhance the knowledge of potential causal links between imaging phenotypes in meningiomas and TERTp mutation status, which is a key molecular marker in the 2021 WHO classification. The combination of 3D FD and lacunarity values independently predicted the TERTp mutation status in grade 2 meningiomas, with an AUC of 0.84. Further, our results show that TERTp-mutant meningiomas have more aggressive imaging features, which can be quantitatively analyzed via fractal features. Our model may be used for preoperative, noninvasive prediction of TERTp mutation status in meningiomas and may allow clinicians to choose a more aggressive surgical approach for TERTp-mutant meningiomas [23]. Moreover, there are currently advances in the development of therapeutic agents to suppress telomerase activity [24], and preoperative prediction of TERTp status may enable the addition of targeted therapy for patients with TERTp mutations in the future.

Among the clinical parameters, older age was the only significant predictor of TERTp mutation in our study. Previous studies have had conflicting results as to whether older age is significantly associated with TERTp-mutant meningiomas; some studies showed that meningioma patients with TERTp mutations were older [4,8], whereas other studies with smaller cohorts did not show statistical significance with respect to age [25,26]. Our result showed that age was a significant factor in predicting TERTp mutation in univariate analysis but not an independent factor n multivariate analysis.

Apart from clinical studies, there has been a relative paucity of studies assessing the radiogenomic characteristics of meningioma in the molecular era [27]. In our study, conventional imaging findings, including tumor location and enhancement pattern, were not significantly different based on TERTp mutation status, which is consistent with the results of a previous study [4]. That meningiomas with TERTp mutations had a larger maximum diameter and higher Ki-67 labeling index may be explained by the higher proliferative potential of TERTp-mutant tumors [28]. TERTp mutation is known to promote cell proliferation and inhibit apoptosis [29]. In other tumor types, such as thyroid and renal cell cancers, TERTp-mutant tumors are larger than TERTp-wildtype tumors, which is consistent with our results [30,31]. Another recent study reported that lower apparent diffusion coefficient values in diffusion-weighted imaging predicted TERTp mutation status in a small number of grade 2 meningiomas. However, advanced imaging sequences such as diffusion-weighted imaging are not routinely performed for meningiomas; therefore, this finding has limited applicability [32]. We used routine T1C sequences and easily accessible tools, which represent a more feasible methodology and may yield reproducible results that can be generalizable in other institutions.

Our study showed that 3D FD and lacunarity are larger in TERTp-mutant meningiomas, suggesting that meningiomas with TERTp mutations exhibit a more complicated texture pattern on MRI than those without TERTp mutations. Previous studies have shown that meningiomas with a high proliferative potential may exhibit highly heterogeneous distributions of proliferating cells, and this heterogeneity may produce irregular shapes [33,34]. TERTp mutation may promote cellular growth and proliferation [35], which can result in an irregular tumor border because of the heterogeneous distribution of proliferating cells [34]. Additionally, the lacunarity of TERTp-mutant meningiomas was higher than that of TERTp-wildtype meningiomas, suggesting that necrosis or cystic changes in the tumor lesion, which are visualized as gaps, may one of the reasons to increase its rotational variance. Although the mechanism underlying the induction of necrosis or cystic change by TERTp mutation is unknown, meningiomas with higher proliferative activity are known to have a higher degree of necrosis [1]. Therefore, TERTp mutation, which promotes increased cellular proliferation, may result in increased necrosis. Previous studies showed that 3D fractal parameters improve the prediction of meningioma grade and aggressiveness, and our study goes further by demonstrating that fractal parameters also reflect the status of molecular markers in meningioma [12,36].

Our study has several limitations. First, this retrospective study from a single institution had a small sample size and included only WHO grade 2 meningiomas. TERTp mutation status was not evaluated in grade 1 meningiomas due to lack of reimbursement in our country. However, as grade 1 meningiomas account for the lowest proportion of TERTp-mutant tumors [4], the number of TERTp-mutant grade 1 meningiomas may not be substantial. Second, there were more TERTp-wildtype tumors than TERTp-mutant tumors. However, the data in our study are in line with those of previous studies reporting proportions of TERTp mutations ranging from 6% to 14% in high-grade meningiomas [3,32]. Third, prognostic markers were not evaluated because patients were recently enrolled. Further studies are needed to assess the correlation of prognostic markers with imaging and genomic features. Despite these limitations, our study identified noninvasive imaging biomarkers to predict TERTp mutation status in line with the new 2021 WHO classification for meningiomas, which is associated with worse prognosis.

In conclusion, 3D FD and lacunarity may be useful imaging biomarkers for predicting TERTp mutation status in grade 2 meningiomas.

## Supporting information

S1 Table Interobserver agreement for qualitative and quantitative imaging analyses. (DOCX)

S1 File Imaging evaluation. (DOCX)

S2 File Fractal analysis by box counting method and surface regularity. (DOCX)
