# Supplementary material for: Three-dimensional fractal dimension and lacunarity features may noninvasively predict TERT promoter mutation status in grade 2 meningiomas
Source: PLoS One. 2022 Oct 20;17(10):e0276342. doi: 10.1371/journal.pone.0276342 (PMC9584385; doi:10.1371/journal.pone.0276342)
Supplement: S1 Table — (DOCX) [file pone.0276342.s001.docx]

**S1 table. Interobserver agreement for qualitative and quantitative imaging analyses.**

| Variables | Interobserver agreement^*^ |
| --- | --- |
| MRI features |  |
| Skull base location | 0.933 (0.832-0.974) |
| Heterogeneous contrast enhancement | 0.891 (0.791-0.991) |
| Capsular enhancement | 0.972 (0.933-0.988) |
| Presence of necrosis | 0.931 (0.891-0.972) |
| Cystic change | 0.872 (0.821-0.923) |
| Skull hyperostosis | 0.970 (0.926-0.988) |
| Skull invasion | 0.898 (0.894-0.899) |
| Max diameter (cm) | 0.990 (0.980-0.999) |
| 3D FD | 0.872 (0.832-0.898) |
| 3D lacunarity | 0.908 (0.894-0.923) |

^*^Data are expressed as either Cohen kappa index or two-way intraclass correlation.
