# Supplementary material for: Three-dimensional fractal dimension and lacunarity features may noninvasively predict TERT promoter mutation status in grade 2 meningiomas
Source: PLoS One. 2022 Oct 20;17(10):e0276342. doi: 10.1371/journal.pone.0276342 (PMC9584385; doi:10.1371/journal.pone.0276342)
Supplement: S1 File — (DOCX) [file pone.0276342.s002.docx]

**Supplementary Material**

**S1. Imaging evaluation**

Tumors arising from the falx, convexity, and ventricles were classified as a non-skull base tumor; all other tumor locations were considered as skull base tumor [1, 2]. Definition of “positive” capsular enhancement was the presence of enhancing layer between the brain and the tumor. Heterogeneous enhancement was defined as qualitative evaluation of intratumoral enhancement and classified as “homogeneous” or “heterogeneous”. In cases of disagreement, the final decision was made through consensus discussion

**References**

1. Kane AJ, Sughrue ME, Rutkowski MJ, Shangari G, Fang S, McDermott MW, et al. Anatomic location is a risk factor for atypical and malignant meningiomas. Cancer. 2011;117(6):1272-8.

2. Park YW, Kim S, Ahn SS, Han K, Kang SG, Chang JH, et al. Magnetic resonance imaging-based 3-dimensional fractal dimension and lacunarity analyses may predict the meningioma grade. Eur Radiol. 2020;30(8):4615-22. doi: 10.1007/s00330-020-06788-8.
