# Supplementary material for: Three-dimensional fractal dimension and lacunarity features may noninvasively predict TERT promoter mutation status in grade 2 meningiomas
Source: PLoS One. 2022 Oct 20;17(10):e0276342. doi: 10.1371/journal.pone.0276342 (PMC9584385; doi:10.1371/journal.pone.0276342)
Supplement: S2 File — (DOCX) [file pone.0276342.s003.docx]

**Supplementary Material**

**S2. Fractal analysis by box counting method and surface regularity.**

Fractal dimension (FD) represents roughness or complexity of binary mask surface. [1] Box counting algorithm is used to calculate FD. [2] A serial grids of increasing boxes are placed over an image in a box counting scan. the number of boxes which are needed to cover the binary mask are counted. Mathematically, FD is then calculated by:

|  | $FD= \lim_{\epsilon\to0} \frac{\log\left( N\left( \epsilon\right) \right)}{\log\left( \frac{1}{\epsilon} \right)},$ | (1) |
| --- | --- | --- |

where $N(\epsilon)$ is the number of counted boxes and $\epsilon$ is a box size and. For empirical estimation of Eq. (1), FD is calculated by dividing the gradient of logarithmic value of count $N\left( \epsilon\right)$ to the gradient of logarithm value of inverse of box size :

|  | ${FD}_{t}= \frac{\log\left( N\left( \epsilon_{t+1} \right) \right)-log(N\left( \epsilon_{t-1} \right))}{\log(\frac{1}{\epsilon_{t+1}})-log(\frac{1}{\epsilon_{t-1}})},$ | (2) |
| --- | --- | --- |

where $\epsilon_{1}$ is the smallest box size 1, $\epsilon_{2}$ is the next bigger box size 2, $\epsilon_{3}$ is the next bigger box size 4, $\epsilon_{4}$ is the next bigger box size 8, $\epsilon_{5}$ is the next bigger box size 16, and so on. For the smallest box size, , $\epsilon_{t-1}$ is set as $\epsilon_{1}$ because there is no previous box size for the smallest box size. Since there is no next box $\epsilon_{t+1}$ for the biggest box size, $\epsilon_{t+1}$ is set as $\epsilon_{t}$. If the 3D binary mask is rougher, higher FD value (slope in log-log plot) is obtained with Eq. (2).

Lacunarity stands for the amount of voids inside the 3D binary mask. [3] To calculate the lacunarity, coefficient of variation (${CV}_{\epsilon}$) are first calculated for the counted boxes which contain a part of 3D binary mask. ${CV}_{\epsilon}$ is calculated by a ratio of standard deviation $\sigma_{\epsilon}$ to mean $\mu_{\epsilon}$ of the pixel intensities inside the box with a size $\epsilon$ as:

|  | ${CV}_{\epsilon}= \frac{\sigma_{\epsilon}}{\mu_{\epsilon}}.$ | (3) |
| --- | --- | --- |

Then, the lacunarity $\lambda_{\epsilon}$ is calculated by the mean of the squares of ${CV}_{\epsilon}$ values as:

|  | $\lambda_{\epsilon}= \frac{1}{N(\epsilon)} \sum_{k} \left( {CV}_{\epsilon}^{k} \right)^{2}.$ | (4) |
| --- | --- | --- |

where ${CV}_{\epsilon}^{1}, {CV}_{\epsilon}^{2}, \ldots, {CV}_{\epsilon}^{K}$ are the ${CV}_{\epsilon}$ values calculated from $K$ counted boxes. The more gaps or holes in the 3D binary mask are, the higher collected ${CV}_{\epsilon}$ values and the higher lacunarity is obtained with the Eq. (4).

**References**

1. Metze K. Fractal dimension of chromatin: potential molecular diagnostic applications for cancer prognosis. Expert review of molecular diagnostics. 2013;13(7):719-35.

2. Falconer K. Fractal geometry: mathematical foundations and applications. John Wiley & Sons; 2004.

3. Plotnick RE, Gardner RH, O'Neill RV. Lacunarity indices as measures of landscape texture. Landscape ecology. 1993;8(3):201-11.
